# Rates of unbalanced chromosome rearrangements associated with pericentric and paracentric inversions: Analysis of molecular chromosome results in embryo samples

**DOI:** 10.1016/j.gimo.2026.104367

**Published:** 2026-02-04

**Authors:** Katrina Merrion, Jessica Adsit, Katherine L. Howard, Anjali D. Zimmer, Jeffrey T. Meltzer, Youbao Sha

**Affiliations:** Natera, Inc, Austin, TX

**Keywords:** Chromosome inversions, Paracentric inversion, Pericentric inversion, Preimplantation genetic testing, Unbalanced chromosome rearrangements

## Abstract

**Purpose:**

To evaluate the outcomes of preimplantation genetic testing for structural rearrangements for individuals with pericentric or paracentric inversions undergoing in vitro fertilization.

**Methods:**

This retrospective study included trophectoderm (TE) embryo biopsy samples analyzed at a single reference laboratory from 37 reproductive couples who underwent 44 in vitro fertilization cycles. TE biopsies and parental samples were genotyped using Illumina CytoSNP-12b microarrays with bioinformatic analysis to determine the parental origin of each chromosome.

**Results:**

For 137 TE biopsy samples with results from 24 individuals with pericentric inversions, the frequency of deletions and duplications related to the inversion was analyzed based on the relative size of the inverted segment. We observed a rate of 5% inversion-associated deletions and duplications for samples from individuals with inversions <30% of the chromosome, 3% from inversions 30% to 50% of the chromosome, and 21% from inversions >50% of the chromosome. Of 43 TE biopsy samples from 7 individuals with paracentric inversions, 2% had inversion-associated deletions and duplications, and 7% had monosomy related to the parental inversion, all of which were from 1 individual.

**Conclusion:**

Although previously considered to have minimal reproductive impact, we identified inversion-associated abnormalities in TE biopsy samples from individuals with paracentric and small pericentric inversions, underscoring a small but meaningful reproductive risk associated with these rearrangements.

## Introduction

Chromosome inversions are structural rearrangements in which a segment is inverted 180 degrees and reinserted into the chromosome. Pericentric inversions involve both chromosome arms and the centromere, whereas paracentric inversions are confined to a single arm and exclude the centromere. During meiosis, crossing over within the inverted segment can lead to the production of chromosomally abnormal gametes, potentially leading to reproductive consequences. Although there are some inversions that carry reproductive risk, there are also common population variants, such as inv(2)(p11.2q13), inv(9)(p11q13), and inv(10)(p11.2q21.2) that have been reported to be benign and not associated with an increased reproductive risk.^1^, ^2^, ^3^

Individuals with a chromosome inversion may produce unbalanced gametes containing terminal deletions and duplications distal to the inverted segment. In individuals with paracentric inversions, unbalanced gametes can also result in embryos with monosomy because of the loss of an acentric chromosome fragment during cell division. Sperm analyses in males with pericentric and paracentric inversions have shown that the frequency of unbalanced gametes ranges from 0% to 38% for pericentric inversions and from 0% to 1% for paracentric inversions.^4^^,^^5^ These studies further suggest that the risk of generating unbalanced gametes increases with the length of the inverted segment.^4^^,^^6^ The highest risk is observed when the inverted segment spans more than 50% of the chromosome, with up to 38% recombinant spermatozoa. A moderate risk is associated with segments between 30% to 50%, yielding up to 2% recombinant spermatozoa, and no significant risk is seen with segments smaller than 30% because no recombinant spermatozoa were identified.^7^^,^^8^ Furthermore, although most paracentric inversions are thought to pose minimal reproductive risk—because of the production of gametes with nonviable acentric or dicentric chromosomes—rare cases of abnormal offspring have been reported.^4^^,^^9^^,^^10^

More recently, next-generation-sequencing-based studies using preimplantation genetic testing for structural rearrangements (PGT-SR) on embryo samples from individuals with paracentric and pericentric inversions have reported unbalanced chromosome rates ranging from 11% to 43%; however, these studies were limited in their ability to determine the parent of origin for the unbalanced chromosome.^11^, ^12^, ^13^ A correlation between the size of the inverted segment and the rate of unbalanced rearrangements has also been observed in PGT-SR studies.^14^

Because most studies on the reproductive impacts of inversions have been confined to sperm studies, evidence of their effects in females and embryos is still emerging. This study provides outcomes of PGT-SR for individuals who underwent in vitro fertilization (IVF) for a known familial pericentric or paracentric inversion, including analysis by parental origin and inversion size.

## Materials and Methods

This retrospective cohort study included all trophectoderm (TE) embryo biopsy samples received at a single reference laboratory between May 2014 and March 2022 from individuals in the United States who pursued IVF with PGT-SR because of a parental chromosome inversion.

Before performing PGT-SR, parental karyotypes were reviewed by a genetic counselor and/or laboratory director to determine if the testing platform was appropriate for testing. For accepted cases, it was confirmed that there was adequate probe coverage to detect the potential unbalanced chromosome products that could result from the parental inversion. IVF procedures and TE biopsies of blastocyst-stage embryos were performed at referring IVF centers. TE biopsy samples and parental samples (blood, buccal, or sperm) were shipped to a single reference laboratory for analysis.

PGT-SR was performed concurrently with PGT for aneuploidy (PGT-A) for all cases. Genotyping was performed using Illumina CytoSNP-12b microarrays with parental support bioinformatic analyses to determine the parental origin of each chromosome, as previously described.^15^ Briefly, parental single-nucleotide variation (SNV) (formerly SNP) data were used to infer expected SNV genotypes in each embryo sample. Observed embryo SNV data were then compared with predicted allele distributions for multiple copy-number hypotheses, and the most likely chromosomal copy-number state was assigned.

The platform detected whole-chromosome and segmental abnormalities, including monosomy, trisomy, or higher-order polysomy, haploidy, triploidy, deletions, duplications, uniparental disomy, and determined the parental origin of chromosome abnormalities. If a chromosome abnormality involved the inversion-associated chromosome but was derived from the parent without the inversion, the event was interpreted as sporadic and unrelated to the parental inversion. Balanced inversions were not detectable with this platform, and the assay has not been validated to distinguish mosaic from nonmosaic abnormalities.

For each IVF/PGT cycle, clinical and demographic data were collected from the test requisition forms, including parental age at testing, history of infertility or recurrent pregnancy loss (RPL), parental karyotype findings, size of the inversion (reported in megabases [Mb] and as a percentage of total chromosome length, categorized as <30%, 30%-50%, or >50%), parent with the inversion, number of TE biopsy samples tested, and genetic testing outcomes. All karyotypes were formatted according to ISCN 2024. This research was reviewed by Salus Institutional Review Board (#19040) and deemed exempt.

Embryo results were classified as euploid if no chromosome imbalance was detected, aneuploid if an imbalance was detected, or No DNA/No Call if there was insufficient DNA for interpretation. Aneuploid results were further categorized to identify cases with an inversion-associated deletion or duplication as a consequence of the parental inversion. In cases of paracentric inversions, an additional subclassification was performed to identify embryos with inversion-associated monosomy originating from the parent with the inversion.

## Results

### Cohort characteristics

**Figure 1:**
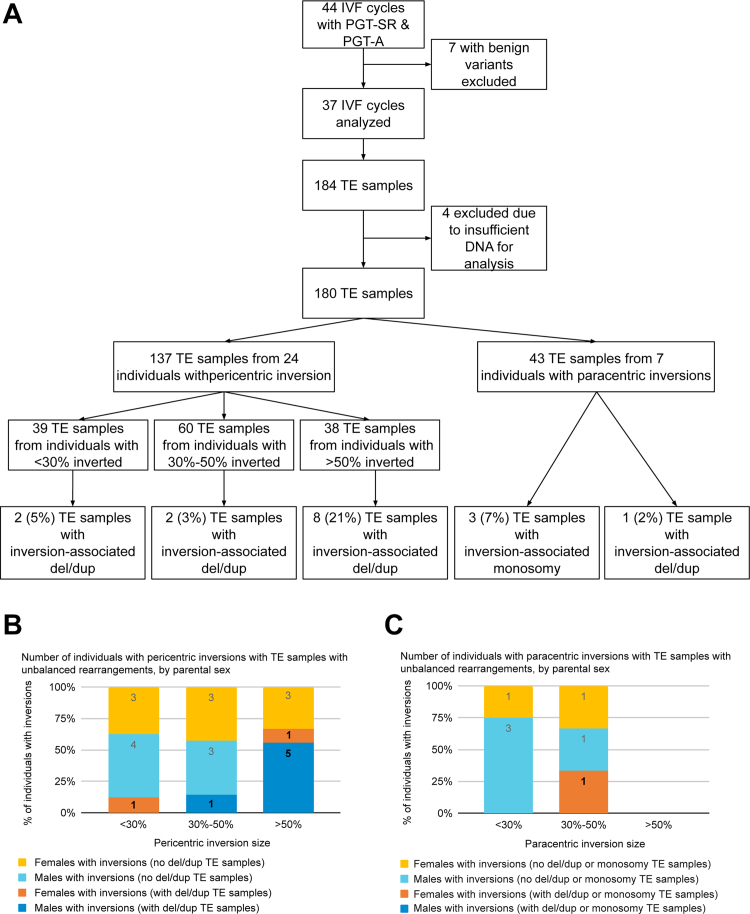
**TE sample results with unbalanced rearrangements.** A. Trophectoderm (TE) biopsy sample results by inversion type and size. B. Bar chart showing the percent of individuals with pericentric inversions with at least 1 TE biopsy sample with an inversion-associated deletion/duplication (del/dup), stratified by inversion size. C. Bar chart showing the percent of individuals with paracentric inversions with at least 1 TE biopsy sample with an inversion-associated deletion/duplication (del/dup) or monosomy, stratified by inversion size.

The cohort consisted of 37 individuals who pursued 44 IVF cycles (Figure 1). Six of these individuals (who pursued 7 IVF cycles) carried benign variants, including inv(2)(p11.2q13), inv(9)(p11q13), and inv(10)(p11.2q21.2). None of these individuals produced embryos with inversion-associated deletions or duplications and were excluded from the remainder of the analysis.

The characteristics of the individuals and their inversions are shown in Supplemental Table 1. Among the 31 individuals, 14 inversions were in females, and 17 were in males (Table 1). The mean age was 35.0 years (range 27-41) for females and 38.8 years (range 30-50) for males with inversions. Although the average parental age in this IVF cohort is higher than the average US reproductive age, the sample size of this study prevented statistical modeling to correct for parental age in the findings. Fifteen individuals (48%) reported a history of RPL, and 4 individuals (13%) reported a history of infertility. Additional reported histories included oligospermia, RPL in a relative with the same inversion, and no or unknown relevant history (Supplemental Table 1).

**Table 1:** TE sample PGT-SR results from individuals with pericentric and paracentric inversions, stratified by inversion size

Inversion Type	Inverted Segment Size (%)	Females w/ inversions (N)	Males w/ inversions (N)	TE Samples Tested (N)	Euploid (N)	Aneuploid (N)	No DNA/No Call (N)	Inversion-Associated Dels/Dups (N)	Inversion-Associated Monosomy (N)
Pericentric	<30%	4	4	39	17	22	0	2	N/A
	30%-50%	3	4	62	26	34	2	2	N/A
	>50%	4	5	39	15	23	1	8	N/A
Paracentric	<30%	1	3	13	7	6	0	0	0
	30%-50%	2	1	31	8	22	1	1	3
Total		14	17	184	73	107	4	13	3

*Dels/Dups*, deletions and duplications; *TE*, trophectoderm.

PGT-SR and PGT-A results were obtained on 180 of 184 (98%) TE biopsy samples. The overall aneuploidy rate in the TE biopsy samples was 59%.

### Pericentric inversions

Twenty-four individuals with pericentric inversions pursued 26 IVF cycles (Figure 1, Table 1). Individuals carrying a pericentric inversion were divided into those with an inverted segment comprising <30%, 30%-50%, and >50% of the chromosome involved.

Of the 137 TE biopsy samples from embryos produced by individuals with pericentric inversions, 39 were from individuals with a <30% inversion, 60 from 30%-50%, and 38 from >50%; inversion-associated deletions and duplications were seen in 5%, 3%, and 21% of TE biopsy samples, respectively (Figure 1A). These TE biopsy samples with inversion-associated deletions and duplications were produced by 1 female with a <30% inversion and history of infertility, 1 male with a 30%-50% inversion and a history of oligospermia, and 6 individuals (1 female and 6 male) with a >50% inversion with histories of RPL, infertility, and oligospermia (Figure 1B).

### Paracentric inversions

Seven individuals with paracentric inversions pursued 11 IVF cycles (Figure 1, Table 1). Of the 43 TE biopsy samples with results, 7% had monosomy associated with the parental inversion, and 2% had inversion-associated deletions and duplications. All TE biopsy samples with abnormal findings were from a single female with an inverted segment comprising 30%-50% of the chromosome. This individual had a family history of RPL in a relative who also carries the inversion.

## Discussion

Although the detrimental effects of chromosome inversions on gamete production and fertility are well documented, most evidence to date derives from studies on sperm from males with inversions. Data on females with inversions and embryo-level outcomes remain limited. This study contributes to the existing literature by analyzing embryos from 31 reproductive couples undergoing IVF with PGT-SR, including both males and females with inversions. Individuals with large pericentric inversions (>50% of the chromosome) exhibited the highest incidence of unbalanced embryos. Notably, however, smaller inversions (30%-50% and <30%) were also associated with unbalanced outcomes, challenging previous assumptions that such inversions pose minimal risk.^7^^,^^8^ In addition, 1 individual in our study with a paracentric inversion produced several embryos with unbalanced recombinants, including deletions and duplications, and monosomy. Overall, these findings support that smaller pericentric inversions and paracentric inversions can confer a measurable risk of unbalanced embryos with important implications for reproductive counseling for individuals with familial inversions.

Our findings contradict previous sperm-based studies suggesting negligible reproductive risks for inversions spanning <30% of the chromosome because those studies did not identify any recombinant spermatozoa in males with inversions of this size.^7^^,^^8^ In our cohort, a female with a <30% pericentric inversion produced embryos with inversion-associated deletions and duplications, consistent with a prior PGT study that reported an unbalanced embryo from a female with a slightly larger (33%) inversion.^16^ Our observation that the individual with a only <30% pericentric inversion in the cohort that produced unbalanced embryos was female also raises the possibility that the origin of the inversion (female vs male) may influence risk. The impact of parental origin has been mixed in the literature, with some studies reporting no effect,^11^^,^^16^^,^^17^ whereas 1 study found a stronger effect associated with paternal origin.^18^ Further research is needed to clarify the role of parental origin on the impact of risk.

Although paracentric inversions are generally considered to confer low reproductive risk because the formation of unbalanced gametes with dicentric or acentric chromosomes is typically not viable, our data show that at least 1 individual with a paracentric inversion produced multiple embryos with unbalanced rearrangements. These findings are consistent with other reports of rare, unbalanced rearrangements,^11^^,^^18^ suggesting that such rearrangements may be more clinically significant than previously assumed. This 1 female with a paracentric inversion in our cohort produced embryos with monosomies, as well as deletions and duplications, highlighting the potential for multiple types of unbalanced rearrangements. This observation, although only of 1 patient, further raises the possibility that the parent of origin may influence outcomes for individuals with paracentric inversions, with female effects remaining less well understood because of the historical reliance on sperm-based studies or case reports of males.^19^

One of the strengths of this study was the inclusion of parental genotype data from SNV microarray analysis with bioinformatics, which enabled us to trace the unbalanced rearrangements to the expected parent of origin. This provided confidence that the observed deletions, duplications, and monosomies were attributed to the parental inversion.

Despite these insights, this study has limitations. The sample size is modest (31 individuals, 37 IVF cycles, and 180 TE biopsies), and we were therefore unable to adjust the data with a model to correct for potential biases, such as maternal age. The platform used does not distinguish between normal and balanced embryos. Additionally, the cohort reflects a population undergoing IVF—often for infertility, RPL, or other indications—which may limit the generalizability of the findings. Data on IVF parameters, such as ovarian stimulation protocols and the number of oocytes retrieved, were not collected, potentially contributing to variability in the number of embryos available for analysis; additionally, pregnancy and birth outcomes were not collected. Lastly, this study was performed on TE biopsy samples which may or may not be compatible with further development; therefore, the reproductive risk described here encompasses both the risk of a liveborn child with an unbalanced karyotype and the risk of pregnancy loss.

Taken together, these findings provide new insights into TE biopsy sample outcomes in both females and males with inversions, indicating that paracentric inversions and even small pericentric inversions still confer some reproductive risk. These results have direct implications for genetic counseling, particularly for individuals considering or undergoing IVF, and underscore the need for future studies in larger, more diverse populations.

## Data Availability

Deidentified data are available in the supplemental material of this article.

## Conflict of Interest

All authors are employees of Natera, Inc, receive salary, and may own stock and/or stock options.
